# Genomic Markers Distinguishing Shiga Toxin-Producing *Escherichia coli*: Insights from Pangenome and Phylogenomic Analyses

**DOI:** 10.3390/pathogens14090862

**Published:** 2025-08-30

**Authors:** Asmaa Elrefaey, Kingsley E. Bentum, Emmanuel Kuufire, Tyric James, Rejoice Nyarku, Viona Osei, Yilkal Woube, Temesgen Samuel, Woubit Abebe

**Affiliations:** 1 Center for Food Animal Health, Food Safety and Defense, Department of Pathobiology, College of Veterinary Medicine, Tuskegee University, Tuskegee, AL 36088, USA; aelrefaey3754@tuskegee.edu (A.E.); kbentum8786@tuskegee.edu (K.E.B.); ekuufire9436@tuskegee.edu (E.K.); tjames2981@tuskegee.edu (T.J.); rnyarku8794@tuskegee.edu (R.N.); vosei3882@tuskegee.edu (V.O.); ywoube@tuskegee.edu (Y.W.); tsamuel@tuskegee.edu (T.S.); 2 Department of Food Hygiene and Control, Faculty of Veterinary Medicine, University of Sadat City, Sadat City 32897, Egypt

**Keywords:** shiga toxin-producing *Escherichia coli* (STEC), pangenome analysis, sequence identity, serogroup-specific markers, core genome phylogeny

## Abstract

Shiga toxin-producing *Escherichia coli* (STEC) are genetically diverse foodborne pathogens of major global public health concerns. Serogroup-level identification is critical for effective surveillance and outbreak control; however, it is often challenged by STEC’s genome plasticity and frequent recombination. In this study, we employed a standardized pangenomic pipeline integrating Roary ILP Bacterial Core Annotation Pipeline (RIBAP) and Panaroo to analyze 160 complete, high-quality STEC genomes representing eight major serogroups at a 95% sequence identity threshold. Candidate serogroup-specific markers were identified using gene presence/absence profiles from RIBAP and Panaroo. Our analysis revealed several high-confidence markers, including metabolic genes (*dgcE*, *fcl_*2, *dmsA*, *hisC*) and surface polysaccharide-related genes (*capD*, *rfbX*, *wzzB*). Comparative pangenomic evaluation showed that RIBAP predicted a larger pangenome size than Panaroo. Additionally, some genomes from the O104:H1, O145:H28, and O45:H2 serotypes clustered outside their expected clades, indicating sporadic serotype misplacements in phylogenetic reconstructions. Functional annotation suggested that most candidate markers are involved in critical processes such as glucose metabolism, lipopolysaccharide biosynthesis, and cell surface assembly. Notably, approximately 22.9% of the identified proteins were annotated as hypothetical. Overall, this study highlights the utility of pangenomic analysis for potential identification of clinically relevant STEC serogroups markers and phylogenetic interpretation. We also note that pangenome analysis could guide the development of more accurate diagnostic and surveillance tools.

## 1. Introduction

Shiga toxin-producing *Escherichia coli* (STEC) are among the most dangerous foodborne pathogens, causing illnesses that range from mild gastrointestinal discomfort to severe, life-threatening complications such as hemolytic uremic syndrome (HUS) [1,2]. The ongoing evolution of these microbes towards increased virulence, combined with diagnostic under-detection and the urgent need for novel and improved therapeutic options, has amplified public health concerns. For instance, in 2010, an estimated 2.8 million STEC infections resulted in 230 deaths, 270 cases of end-stage renal disease (ESRD), and 3890 cases of HUS [3]. To date, more than 470 STEC serotypes have been identified [4,5].

Although *Escherichia coli* O157:H7 remains the most commonly associated serotype with human infections, an increasing number of foodborne outbreaks have been linked to non-O157 serogroups, including O26, O45, O103, O111, O121, O145, and O104 [6,7]. The distribution of these serogroups varies by geographic region. In North America, most reported STEC infections are caused by serogroups O26, O45, O103, O111, O121, O145, and O157, collectively known as the “top seven” [8,9]. Between 2000 and 2010, 83% of non-O157 STEC infections in the United States were attributed to the “big six” non-O157 serogroups, O26, O111, O103, O121, O45, and O145 [9,10].

In 2011, *E. coli* O104:H4 emerged as a causative agent of a large outbreak across 16 European countries, primarily in Germany, with several travel-associated cases reported in North America [11]. The O104:H4 serotype is not classified as a typical O157-like STEC. Instead, it represents a hybrid pathotype, exhibiting virulence determinants characteristic of both STEC and enteroaggregative *E. coli* (EAEC) [12]. O104:H4 typically exhibits an EAEC backbone (*aggR*^+^, *pAA*^+^) with acquisition of an *stx2* encoding prophage. Characteristically, EAEC strains carry the aggregative-adherence plasmid (pAA), which encodes the AraC-family regulator AggR (*aggR* gene), a widely used molecular marker of the enteroaggregative phenotypes. AggR activates transcription of hallmark EAEC loci, including aggregative adherence fimbriae (AAF, *agg*/*aaf*), the antiaggregation protein Dispersin (*aap*) together with its secretion system (*aatPABCD* transporter), and components of the *aai* type VI secretion apparatus [13]. According to Rahal et al. [14], this event underscores the significance of non-traditional serotypes in public health. Notably, while most STEC serotypes are associated with ruminant reservoirs, *E. coli* O104:H4 appears to persist within human populations and lacks a known animal reservoir [15].

Culture-based methodologies for STEC detection, such as sorbitol-MacConkey agar, are cost-effective but have notable drawbacks, including false-negative due to the emergence of sorbitol-fermenting non-O157 and O157 STEC serotypes [16]. Similarly, antigen agglutination test with specific antisera against O- and H-antigens of *E. coli* are time-consuming and prone to inaccuracies [17,18]. To overcome these limitations, recent approaches increasingly employ molecular serotyping, now routinely complemented by genome-based approaches such as in-silico serotyping from WGS assemblies [19,20]. Additional complementary strategies include Achtman MLST/cgMLST via EnteroBase [21], and recombination-aware core-genome phylogeny using tools like Gubbins/ClonalFrameML v1.12 [21,22].

As noted by Chaudhuri and Henderson [23], STEC possesses a highly dynamic genomic architecture consisting of a conserved core genome and a variable accessory genome rich in phages, plasmids, and pathogenicity islands. A deeper understanding of these genomic dynamics is essential to developing improved diagnostic tools, identifying novel genetic markers, and designing more effective intervention strategies. Pangenome analysis and whole-genome sequencing (WGS) techniques have revolutionized bacterial genomics by enabling high-resolution analysis of genetic diversity both within and across species [24]. The pangenome approach, which assesses the full complement genes across strains of a species, is a powerful strategy for differentiating between core and accessory genomic elements [25].

In *E. coli*, pangenome studies have identified an expansive gene repertoire of approximately 89,000 genes, in contrast to a relatively small core genome of around 3100 genes [26]. However, most of this research has been conducted at the species level [27]. Comparative analysis across sequence types (STs) or serogroups can offer additional insights into the evolutionary dynamics of *E. coli*. Inter-pangenome comparisons can reveal genes that are core within one group but variable or absent in others, shedding light on differential selective pressures and adaptation mechanisms unique to specific serogroup [28]. This is particularly relevant for STEC, while different serotypes are associated with distinct clinical outcomes and antigenic profiles [29]. Although O157 remains a major concern, non-O157 STEC serogroups contribute significantly to the global disease burden [7,30]. The high genetic diversity of *E. coli* driven largely by horizontal gene transfer is central to its adaptability and persistence [31,32]. This diversity also presents opportunities to advance our understanding of the pathogen beyond the species level. Investigating the core and accessory genomes of various STEC serogroups can facilitate the identification of conserved genetic elements critical for broad-spectrum detection and serogroups-specific markers suitable for high-resolution typing.

In this study, we conducted a comparative pangenomes analysis of STEC serotypes to identify genetic elements that could serve as the basis for serotype-specific identification. The analyzed serogroups included the “top seven” most frequently reported in North American surveillance, as well as the emergent O104:H4 serotype from Europe. Two pangenome analysis tools, RIBAP and Panaroo, were used to explore the genetic diversity of these eight STEC serogroups and to identify unique genetic targets associated with each serogroup.

## 2. Materials and Methods

### 2.1. Genomic Dataset Collection and Quality Filtering

On 3 January 2025, whole genome assemblies of 160 Shiga toxin-producing *Escherichia coli* (STEC) were retrieved from the National Center for Biotechnology Information (NCBI) database in nucleotide FASTA format. The dataset consisted of 20 genome assemblies for each of the eight predominant serogroups O157, O145, O121, O111, O103, O26, O45, and O104.

Each genome assembly was assessed for quality using CheckM (v1.2.3) [33], and met our inclusion requirements with >95% completeness and <5% contamination for further analysis. The total number of genomes included in this study was capped at 160 due to the substantial disk space and computational resources required to perform pairwise gene comparisons and subsequent integer linear programming (ILP) optimization using RIBAP, particularly for datasets exceeding 100 genomes [34]. NCBI accession numbers for all genome assemblies used in this study are provided in Supplementary Table S1.

### 2.2. Genome Annotation and Pangenome Analysis

Genome annotation was performed using Prokka (v1.14.6) [35,36] via a Nextflow platform integrated within RIBAP. The use of Nextflow enabled standardized gene prediction and functional annotation across all datasets, ensuring reproducibility of the analyses pipeline [37].

Following annotation, the resulting GFF3 files were used as input for comprehension pangenome analysis using both Panaroo (v1.2.8) and RIBAP (v1.1.0). Panaroo, a graph-based pangenome clustering tool [38], operates using protein sequence data, while RIBAP employes an integer linear programming approach to refine gene clusters initially predicted by Roary, thereby improving core genes identification [34].

Both tools were executed using a 95% sequence identity threshold to ensure a high-stringency clustering, reduce the influence of fragmented annotations, and minimize spurious gene calls. This consistent threshold also helped mitigate potential biases in each tool’s ability to identify putative serogroup-specific markers.

Virulence genes, including Shiga toxin *(stx1*/*stx2* and their subtypes), the aggregative adherence regulator (*AggR*; gene *aggR*) and intimin (*eae*), were detected by screening genome assemblies against the Virulence Factor Database (VFDB) using ABRicate, applying thresholds of ≥90% sequence identity and ≥60% coverage [39,40].

A core-genome-based phylogenetic tree was generated as part of the RIBAP analysis and was subsequently used to map serotype positions of the isolates by using iTOL v6 [41].

Three discordant isolates-showing serotype–phylogeny mismatch in which the assigned O:H serotype did not cluster with its corresponding serotype clade in the core-genome phylogenetic tree were re-analyzed using a multi-step genomic pipeline. Serotype-Finder v2.0 [20] was used to confirm the O- and H-antigen types for each isolate, applying thresholds of ≥90% sequence identity and ≥60% coverage. Sequence types (STs) were assigned based on the Achtman MLST scheme using mlst software v 2.23 [42]. Whole-genome relatedness to references strains (listed in Supplementary Table S3) was assessed using FastANI v1.34 [43] and Mash v2.3 [44]. To evaluate O-antigen gene cluster (OAGC) similarity, the *galF*–*gnd* region, which brackets the OAGC in most *E. coli* was extracted in silico from each genome using samtools faidx v 1.22 [45] to enable locus comparison, aligned with MAFFT v7.526 [46], and pairwise distances were computed using the Kimura 2-parameter model in EMBOSS distmat [47]. Using these conserved flanks as boundaries enabled capture of the complete *wzx*/*wzy* (Wzy-dependent) or *wzm*/*wzt* (ABC-transporter) module along with adjacent glycosyltransferase and sugar-pathway genes. Together, these elements constitute the core genetic unit for assessing O-antigen similarity and detecting potential serogroup switching events [48].

### 2.3. Identification, Validation, and Cross-Verification of Candidate Marker Genes

Candidate serogroup-specific markers were initially identified from the gene presence/absence profiles generated by RIBAP and Panaroo. Genes that were both consistently present across all genomes within a given serogroup and absent from others were selected as preliminary candidates. These genes were then subjected to a multi-step validation process.

First, each candidate gene was analyzed using BLASTN against the NCBI nucleotide database (https://blast.ncbi.nlm.nih.gov/Blast.cgi, accessed on 15 January 2025) to assess specificity, sequence coverage, nucleotide identity, and potential cross-reactivity with non-target genomes. Subsequently, the presence or absence of each gene was further verified in the curated genome set using Geneious Prime (v2024.0.1) (https://www.geneious.com). Cross-validation was then performed by confirming that each candidate gene consistently appeared in the gene presence/absence matrices generated by both RIBAP and Panaroo, thereby improving marker reliability.

Additionally, the core-genome phylogenetic tree from the RIBAP output and the genes presence/absence matrices from both tools were compared and visualized using Phandango [49].

### 2.4. Functional Characterization of Serogroup-Specific Genes via Gene Ontology Annotation

To investigate the functional roles of serogroup-associated genetic targets, Gene Ontology (GO) annotation was performed using the UniProt database (release 2024_06, accessed on 20 January 2025).

### 2.5. Visualization of Figures

**Figure 1:**
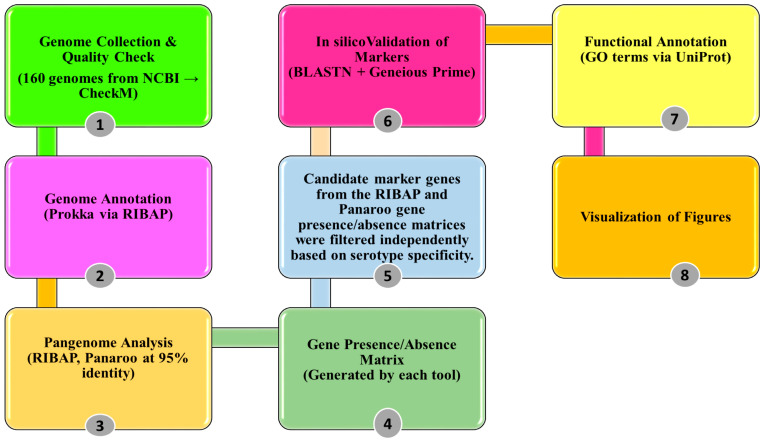
Workflow of Methodology.

All visualization, including heatmaps, were generated using R software (v 4.3.1) with the following packages ggplot2 [50], ggforce [51] and viridis [52]. A summarized workflow of methodology is provided in Figure 1.

Summarized workflow for STEC marker identification: integrating pangenome analysis, candidate marker validation, and functional classification.

## 3. Results

### 3.1. Comparative Pangenome Composition and Genomic Diversity of STEC Using RIBAP and Panaroo Tools

**Figure 2:**
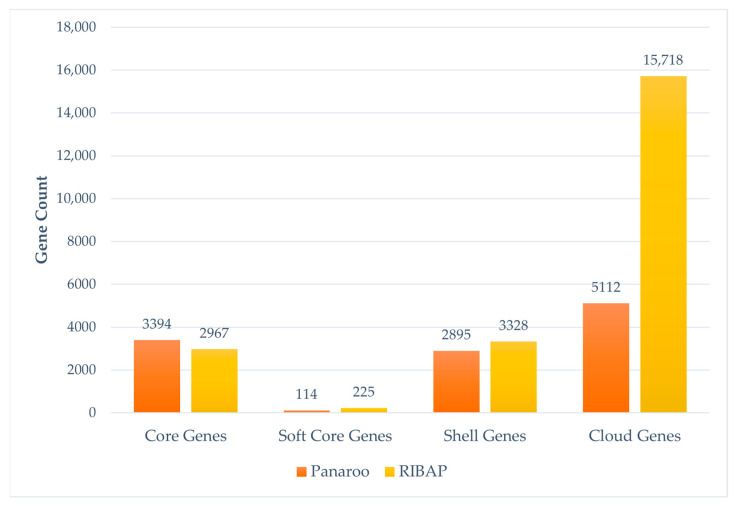
Gene composition identified by pangenome tools in STEC genomes of eight serogroups. Bar plots compare the distribution of core, soft core, shell, and cloud genes identified by RIBAP and Panaroo. The figure illustrates differences in gene categorization and total gene counts among the tools.

Pangenome analysis of the 160 STEC genomes using both RIBAP and Panaroo revealed an open pangenome structure, characterized by a substantially larger accessory genome than the core genome. This indicates significant genetic variability among STEC serotypes, as illustrated in Figure 2.

**Figure 3:**
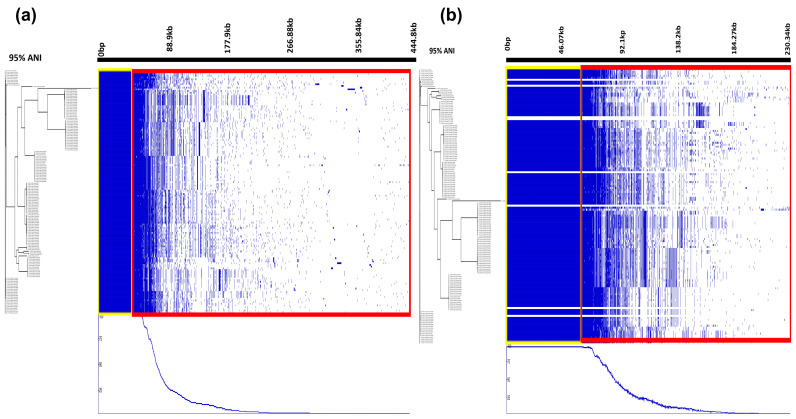
Pangenome distribution and phylogenetic relationships of Shiga toxin-producing *Escherichia coli* (STEC) serotypes analyzed using RIBAP (**a**) and Panaroo (**b**). Core gene-based phylogenetic trees (**left**) illustrate the evolutionary relationships among ten STEC serotypes. Gene presence and absence are represented in blue and white, respectively. Yellow rectangles denote conserved core genome regions conserved at a 95% sequence threshold, while the red rectangles highlight accessory genome regions.

Marked differences were observed in gene category distribution and total gene counts across the two tools (Figure 3). Due to graph-based error correction and stricter filtering, Panaroo identified 11,515 gene clusters significantly fewer than 22,238 clusters detected by RIBAP.

Panaroo reported a core genome of 3394 genes, which was higher than the 2967 core genes identified by RIBAP (Figure 2). Additionally, Panaroo’s stringiest criteria resulted in fewer soft-core genes (present in 95–99% of genomes)—114 genes, compared to 225 identified by RIBAP.

RIBAP identified 3328 shell genes (genes present in 15–95% of genomes), while Panaroo reported 2895 identified (Figure 3).

A notable pattern in the gene presence/absence matrix was that, unlike RIBAP, Panaroo’s output exhibited a fractionated profile, seen as white streaks extending from the core to accessory gene segment (Figure 2).

**Figure 4:**
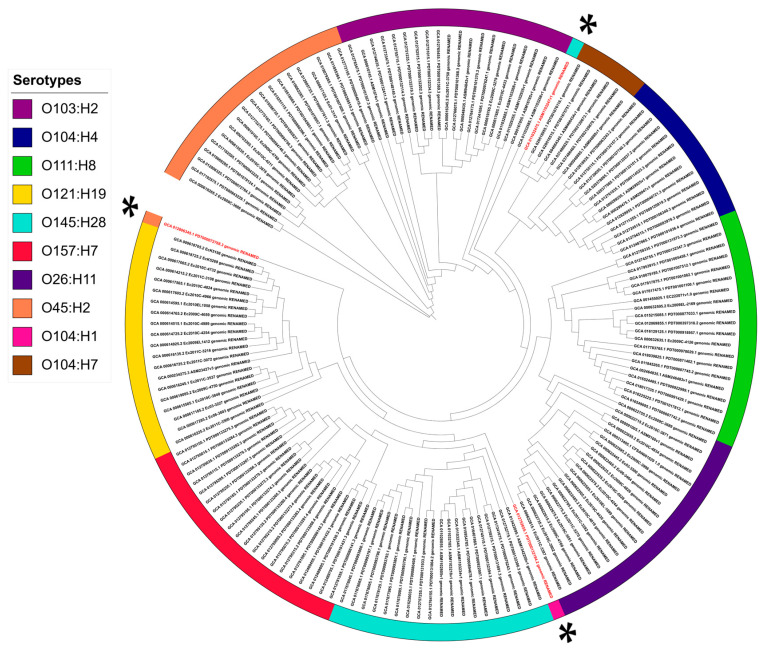
Core–genome phylogeny of STEC isolates across ten serotypes. Maximum-likelihood tree constructed from RIBAP-derived core–gene alignments. Colored outer strips correspond to serotype predictions (legend, **left**), illustrating the phylogenetic distribution of ten STEC serotypes. Red-labeled isolates and asterisks (*) indicate genomes that do not cluster with their expected serotype clade.

Moreover, three genomes were positioned outside their expected serotype clade. For example, one genome of O145:H28 isolates clustered within the O104:H7 lineage, one O104:H1 genome nested within the O157:H7 clade away from O104:H4 and O104:H7 clades, and individual genome of O45:H2 was clustered in a clade near the O121:H19/O157:H7 clade as shown in (Figure 4).

### 3.2. Identification and Validation of Specific Genetic Markers for Major Shiga Toxin-Producing *Escherichia coli* Serogroups

A total of 48 candidate genetic markers were identified as serogroup-specific for the major Shiga toxin-producing *Escherichia coli* (STEC) serogroups, as summarized in Table 1. For each serogroup, two to thirteen unique genes were selected from the presence/absence matrices and subsequently validated.

BLASTN analysis of the candidate genes demonstrated high specificity. Most markers achieved 100% sequence identity and coverage, with a little of cross-reactivity against non-target serogroups or unrelated bacterial taxa (Supplementary Table S2).

### 3.3. Functional Characterization of Serogroup-Specific Genes via Gene Ontology Annotation

**Figure 5:**
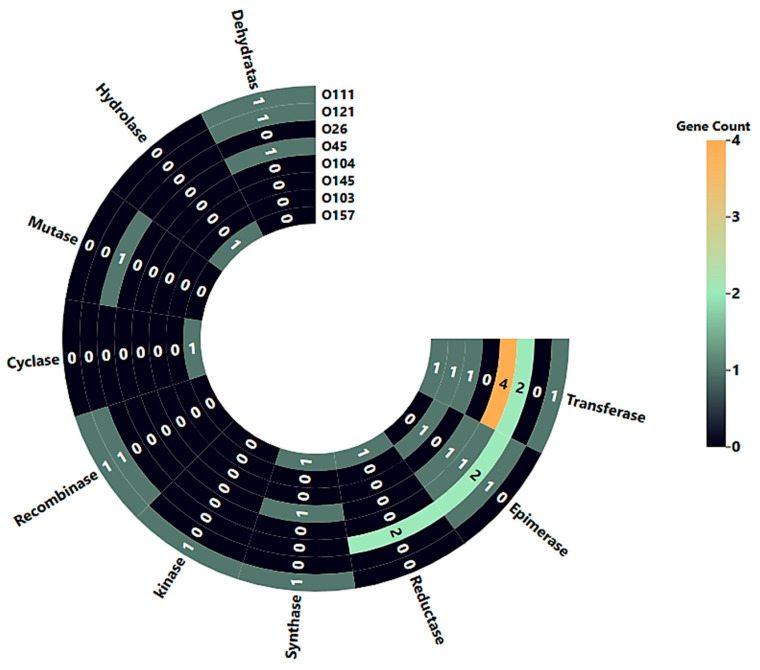
Enzyme classification of serogroup-specific genes based on UniProt Gene Ontology. Radial heatmap Illustrating the distribution of enzyme types among the 31-serotype specific proteins identified across the eight STEC serogroups.

Gene Ontology (GO) annotation using the UniProt database revealed that 31 out of the 48 serogroup-specific candidate genes encode enzymes. These enzymes were classified by molecular function into various categories, including transferases (10/31), epimerases (6/31), reductases (3/31), synthase (3/31), kinase (1/31), recombinase (2/31), cyclase (1/31), mutase (1/31), hydrolase (1/31), and dehydratases (3/31), as illustrated in Figure 5.

Subcellular localization predictions indicate that most enzymes were cytoplasmic, while several were membrane-associated. Notably, key membrane-targeted proteins included *WecA_1*, *chiP_1*, *dgcE_2*, *dmsA_1*, *pglJ*, *yehC 1*, *rfbX*, *wzc*, and *wzzB* (see Table 1).

**Figure 6:**
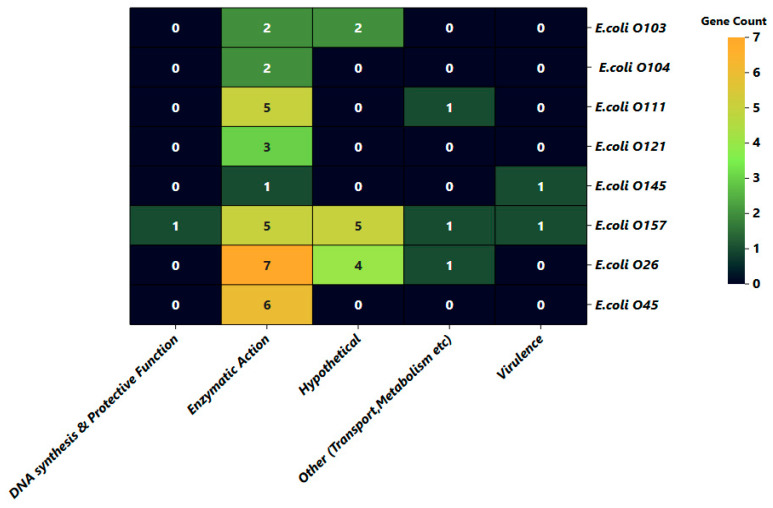
Functional distribution of identified serogroup markers across the eight shiga toxin-producing *E. coli* (STEC) serogroups. The heatmap displays the distribution of identified serotype-specific genes across five major functional categories: DNA synthesis and protection, enzymatic activity, hypothetical proteins, transport/metabolism, and virulence. Color intensity reflects gene abundance, with lighter shades indicating higher counts and darker shades representing lower counts. Numeric values within each cell denote the total number of genes assigned to a specific functional category for each serogroup.

Additionally, the 48 identified genes were categorized into five major functional groups based on their biological roles, as shown in Figure 6. It is noteworthy that 11 out of 48 (22.91%) of the identified proteins were annotated as hypothetical.

## 4. Discussion

Pangenome analysis has entered a new era of complexity, driven by the availability of thousands of complete genomic sequences from diverse strains of the same species [53]. In this study, we conducted a comprehensive pangenome analysis of 160 shiga toxin-producing *Escherichia coli* (STEC) genomes, encompassing eight clinically important serogroups. The aim was to identify promising genetic markers for serogroup-specific identification and to characterize the overall pangenome structure of STEC.

Our findings revealed that RIBAP was significantly more computationally demanding than Panaroo, largely due to its pairwise comparison method that employs integer linear programming (ILP). As previously reported by Lamkiewicz et al. [34], analyzing even 71 genomes with RIBAP can require up to 3.4 TB of disk space. This resource-intense nature posed a limitation in our study, which involved a considerably larger dataset of 160 genomes.

When comparing the pangenome matrices generated by Panaroo and RIBAP, we observed that Panaroo’s matrix exhibited distinct white streaks for certain genomes, an indication of missing gene calls or data filtering. This phenomenon is likely due to Panaroo’s stringent graph-based quality control measures, which eliminates coding sequences with frameshifts, implausible gene lengths, or assemblies with gene/contig-statistics falling outside the interquartile range of the dataset. This filtering occurs even when assemblies meet CheckM’s thresholds for ≥95% completeness and low contamination [33,38].

Despite these discrepancies, we found that the same genomes remained represented in RIBAP’s matrix. This may be attributed to RIBAP’s ILP-based refinement approach, which aims to resolve fragmented or misannotated genes and incorporate them into a reconciled pangenome profile [34], albeit at the cost of potential annotation noise and increased computational burden.

The tools used in this study, RIBAP and Panaroo differed significantly in their representation of accessory genomes, definition of core genes, and estimated total pangenome size. Panaroo produced a larger and more inclusive core genome, likely due to its graph-based filtering and alignment correction strategies, which prioritize conserved orthologs and eliminate spurious gene calls or collapsed paralogs [38]. In contrast, RIBAP applies a gene-by-gene progressive alignment strategy with stricter orthology criteria. This conservative approach results in a smaller core genome and a larger cloud gene set by assigning genes with minor sequence variations to separate clusters [34].

Despite their methodological differences, both tools consistently confirmed the open nature of the STEC pangenome characterized by a continually expanding accessory genome that exceeds the size of the core genome. This pattern reflects the high genomic plasticity and extensive horizontal gene transfer of *E. coli* [54] and aligns with findings from prior studies [55]. Comparatively, while Salmonella enterica genetically close to *E. coli,* species-level studies suggest it tends towards closed pangenome. However, at the serotype level, *Salmonella* also demonstrates an open pan-genome structure [56,57,58]. This contrast is attributed to the rapid depletion of the cross-serovar accessory gene pool in Salmonella enterica’s, where horizontal gene transfer predominantly occurs within serovars rather than between them, resulting in a limited number of novel genes per genome relative to genome size [56,57,58].

The remarkable genomic plasticity of STEC was further highlighted by three isolates in our dataset that failed to cluster within their expected serotype clades, leading to phylogenetic discordance (Figure 4). In silico serotyping using SerotypeFinder v2.0 (≥90% identity, ≥60% coverage) confirmed that the assigned metadata matched the predicted, serotype for all genomes, except one isolate originally identified as O145:H28, which was reassigned as O121:H7. The complete O:H serotypes for all isolates are reported in Supplementary Table S1. Additionally, one O104:H1 isolate formed a distinct lineage separate from the O104:H4/H7 cluster, which is consistent with the fact that serotypes are defined by both O and H antigens; differences in H types often reflect distinct genomic lineages within the same O group.

Furthermore, one O45:H2 isolate (ST306) clustered outside the main O45:H2 clade despite concordant O and H serogroups. Its O-antigen cluster (*galF–gnd*) was nearly identical to the O45:H2 reference (Kimura ≈ 0.01), yet genome-wide similarity to references was only intermediate (FastANI ≈ 98.6%; Mash ≈ 0.012–0.013), suggesting within-serotype diversity or recombination. The re-typed O121:H7 isolate clustered with O104:H7 (FastANI = 99.38%; Mash = 0.0071) and carried an O121 O-antigen locus (Kimura = 0.14), consistent with O-antigen exchange and metadata mis-serotyping. Collectively, these findings illustrate that apparent serotype–phylogeny incongruence within a shared O-serogroup may arise from different H-antigen types, recombination, and O-antigen gene cluster (OAGC) exchange, resulting in mosaic and atypical isolates. This underscores the need to interpret serotype in the context of complete O:H antigens [59,60] (Supplementary Table S3). Similar incongruences have been documented in comparative *E. coli* studies, where lateral gene transfer disrupts the correlation between serotypes and phylogeny [61,62], highlighting OAGC mobility of as a major driver of STEC diversity.

In this study, virulence determinants were characterized to document *stx* types/subtypes and *eae* status for each genome (Supplementary Table S1). Clear lineage-specific patterns were observed: O26:H11 and O103:H2 were predominantly *eae*^+^, *stx1*^+^ (with occasional *stx1* + *stx2*); O111:H8 was mostly *eae*^+^ with *stx1* or *stx1* + *stx2*; O121:H19 was uniformly *eae*^+^ carrying *stx2* or *stx1*; O145:H28 generally carried *eae* with *stx2*, *stx1* or both; and O157:H7 was typically *eae*^+^, *stx2*^+^ (occasionally with *stx1*).

By contrast, O104 serogroup genomes were typically *eae*^−^, with subsets carrying *stx2*^+^ together with *aggR*^+^ or being *stx*^−^/*aggR*^−^, consistence with EAEC–STEC hybrid backgrounds [63]. Rarer *eae*^+^/*stx*^−^ isolates likely reflect loss of the *stx*-converting prophages, while *eae*^−^/*stx*^+^ combinations represents locus of enterocyte effacement (LEE)–negative STEC [64]. Across O104: H types, *aggR* was detected in (11/13) O104:H4 genomes but was absent in O104:H7/H1, similar to the O145:H28 isolate, which clustered within the O104:H7 clade and was likewise *aggR*^−^. These patterns emphasize that mobile loci frequently decouple virulence profile from serotype.

Consequently, accurate detection of highly pathogenic STEC in food remains a major diagnostic challenge. Conventional culture-based diagnostic methods are labor-intensive and time-consuming, reinforcing the need for rapid and robust molecular detection approaches [65]. Such strategies could be strengthened by targeting additional genes more specifically associated with enterohemorrhagic *E. coli* (EHEC), particularly those simultaneously harboring *stx* and *eae* [66].

The key objective of this study was to identify and characterize serogroup-specific genetic markers across the eight major STEC serogroups. For instance, *dmsA* encodes a dimethyl sulfoxide (DMSO) reductase subunit involved in anaerobic respiration by converting DMSO to dimethyl sulfide [67]. *fcl_2* is involved in the synthesis of GDP-fucose, a sugar crucial for O-antigens biosynthesis and surface polysaccharide variation, both of which contribute to immune evasion and serotype differentiation [68]. *DgcE*, which synthesizes cyclic-di-GMP, plays a regulatory role in motility, biofilm formation, and stress response, key attributes for the prolonged colonization and persistence of O157 strains in host environment [69]. Collectively, these genes reflect lineage-specific metabolic adaptations consistent with previously reported functional trait in STEC O157 serogroup.

Additionally, several genes involved in O-antigen biosynthesis and capsule modification were shown to vary by serogroup. *FdtB*, which contributes to the synthesis of dTDP-3-amino-3,6-dideoxy-D-galactose, was uniquely conserved in O103 serogroup, corroborating structural studies of O103-specific O-antigen sugar modifications [70]. Similarly, *hisC* and various capsule acetyltransferases were consistently identified in O145 serogroup, supporting the view that metabolic and capsule-associated genes are critical for molecular serotyping [71]. Other serogroup-specific genes with roles in O-antigen synthesis and transport included *rfbX* (*wzx*), *wzzB*, and *capD*. Specifically, *wzzB* regulates O-antigen chain length, thereby influencing lipopolysaccharide (LPS) structure and immune recognition [72,73]; *capD* is involved in capsule polysaccharides assembly [74], and *rfbX* encodes an O-unit flippase essential for O-antigen export [75].

Functional annotation of these serogroup-specific markers highlighted biological processes central to STEC pathogenicity, including O-antigen production, carbohydrate metabolism, and surface structure assembly. Several genes, such as *galU*, *gmd*, and *rfbC*, were associated with nucleotide sugar biosynthesis. In addition, stress response and recombination related genes, including *pinR*, parE4, and *xerC,* were identified. As noted by Sampaio et al. [76], these findings underscore the role of genome plasticity in STEC’s long term evolution and ecological adaptability.

The observed diversity in virulence and transport-associated genes among serogroups suggest distinct ecological niches and pathogenic pathways. Notably, over 22% of the identified markers were annotated as hypothetical proteins, representing a substantial subset of genes with currently unknown functions. These uncharacterized proteins may play important, yet unexplored roles in STEC virulence, environmental persistence, or resistance mechanisms, and thus represent promising targets for future functional studies.

A limitation of this study is that it focused primarily on well-established and known STEC serotypes, including the “big six” [77]. While some of these serotypes may currently be rare, their inclusion provided valuable insights into pangenome dynamic, and they may also re-emerge as public health threats in the future. Emerging serotypes such as O80:H2 [78] and O177:H11/O177:H25 [79] further highlight the need to expand genomic investigation beyond the traditional serotypes. Future studies will aim to characterize these emerging lineages for genomic markers, as demonstrated here, to support rapid and specific detection. Such efforts have the potential to generate novel perspectives and uncover new research insights.

## 5. Conclusions

Using a cross-validated analytical framework, this study identified high-confidence serogroup-specific gene markers that may serve as candidates for future investigation into novel virulence mechanisms or for enhancing molecular diagnostic strategies. The analysis also highlighted substantial genetic diversities among STEC isolates, with discordant phylogenetic placements largely attributable to differences in H-serogroups. Collectively, these findings advance our understanding of STEC population structure and provide a foundation for the development of improved detection and diagnostic approaches.

## Figures and Tables

**Table 1:** Candidate markers for eight key STEC serogroups, with accession numbers, sequence lengths, and functional gene categories.

Serogroups	Protein Name (Gene)	Accession No.	Size (bp)	Subcellular Localization	Functional Gene Category
**O157**	Hypothetical protein	AAG58872.1	624	Unknown	Hypothetical
	Hypothetical protein	WP_000369315.1	672	Unknown	Hypothetical
	Hypothetical protein	EHV01783.1	135	Unknown	Hypothetical
	Hypothetical protein	WP_000526425.1	300	Unknown	Hypothetical
	YehF protein	WP_001215588.	729	Unknown	Hypothetical
	GDP-L-fucose synthase (*fcl_2*)	AAC32346.1	966	Cytoplasm	Enzymatic action
	GDP-mannose mannosyl hydrolase (*gmm_2*)	WP_000478513.1	510	Cytoplasm	Enzymatic action
	Mannose-1-phosphate guanylyltransferase 1(*manC*1*_2*)	WP_001278239.1	1449	Cytoplasm	Enzymatic action
	Putative diguanylate cyclase DgcE (*dgcE_2*)	ASL58608.1	2808	Inner cell membrane (cytosolic side)	Enzymatic Action
	Chitoporin (*chiP_1*)	ACI72635.1	480	Outer membrane (porin)	Others (Transport, Metabolism, etc.)
	Dimethyl sulfoxide reductase (*dmsA_1*)	WP_000380694.1	2382	Periplasmic side of the inner cell membrane	Enzymatic action
	Toxin ParE4	WP_000277484.1	282	Cytoplasm	Virulence
	Protein YciF	AAG56012.1	501	Cytoplasm	DNA synthesis and protection
**O103**	Hypothetical protein	WP_001064117.1	407	Unknown	Hypothetical
	Hypothetical protein	EJV1286191.1	528	Unknown	Hypothetical
	UDP-glucose 4-epimerase (*lnpD*)	WP_000996555.1	1029	Cytoplasm	Enzymatic action
	N-acetylgalactosamine-N, N’-diacetylbacillosaminyl-diphospho-undecaprenol4-alpha-N-acetylgalactosaminyltransferase(*pglJ*)	HGU3025143.1	1092	Membrane-associated	Enzymatic action
**O145**	Putative fimbrial chaperone YehC (*yehC 1*)	EKO1170350.1	292	Periplasmic space (inner-membrane-anchored chaperone)	Virulence
	Histidinol-phosphate aminotransferase (*hisC*)	WP_001099213.1	1071	Cytoplasm	Enzymatic action
**O104**	UDP-N-acetylglucosamine 2-epimerase (*neuC*)	WP_000723247.1	1164	Cytoplasm	Enzymatic action
	CMP-N, N’-diacetyllegionaminic acid synthase (*Legl*)	EGT66549.1	330	Cytoplasm	Enzymatic action
**O45**	Serine acetyltransferase (*cysE_1*)	EOV7831118.1	240	Cytoplasm	Enzymatic action
	dTDP-4-dehydrorhamnose 3,5-epimerase (*rfbC*)	WP_001100944.1	540	Cytoplasm	Enzymatic action
	UDP-N-acetyl-alpha-D-glucosamine C6 dehydratase (*pglF*)	WP_001435027.1	1887	Cytoplasm	Enzymatic action
	UDP-N-acetylbacillosamine N-acetyltransferase (*pglD*)	WP_000342233.1	561	Cytoplasm	Enzymatic action
	Undecaprenyl-phosphate alpha-N-acetylglucosaminyl 1-phosphate transferase (wecA_*1*)	WP_000966114.1	1035	Inner cell membrane	Enzymatic action
	Glucose-1-phosphate thymidylyltransferase 2 (*rffH_1*)	WP_000676072.1	873	Cytoplasm	Enzymatic action
**O26**	Hypothetical protein	AOM43287.1	508	Unknown	Hypothetical
	UDP-23-diacetamido-23-dideoxy-D-glucuronate 2-epimerase (*wbpI*)	WP_000734421.1	1131	Cytoplasm	Enzymatic action
	UDP-2-acetamido-26-beta-L-arabino-hexul-4-ose reductase (*wbjC*)	WP_001429673.1	1107	Cytoplasm	Enzymatic action
	UDP-glucose 4-epimerase (*capD*)	WP_000475914.1	1035	Cytoplasm	Enzymatic action
	hypothetical protein	EET7051301.1	795	Unknown	Hypothetical
	hypothetical protein	WP_000291456.1	1023	Unknown	Hypothetical
	Putative O-antigen transporter (*rfbX*)	WP_000914145.1	1263	Inner cell membrane	Others (Transport, Metabolism, etc.)
	Glucose-1-phosphate thymidylyltransferase 1 (*rfbA*)	WP_000857547.1	879	Cytoplasm	Enzymatic action
	dTDP-4-dehydrorhamnose reductase (*rfbD*)	WP_001023633.1	900	Cytoplasm	Enzymatic action
	hypothetical protein	WP_001116073.1	1214	Unknown	Hypothetical
	D-inositol-3-phosphate glycosyltransferase (*mshA_1*)	WP_000862644.1	1041	Cytoplasm	Enzymatic action
	Phosphomannomutase/phosphoglucomutase (*algC*)	KGM70217.1	836	Cytoplasm	Enzymatic action
**O121**	dTDP-glucose 4,6-dehydratase (*rfbB*)	KDV82622.1	712	Cytoplasm	Enzymatic action
	Tyrosine recombinase (*xerC_1*)	WP_001234104.1	1212	Cytoplasm	Enzymatic action
	dTDP-4-keto-6-deoxy-D-glucose 3,5-epimerase (*wbtF*)	EFF6995043.1	1116	Cytoplasm	Enzymatic action
**O111**	GDP-mannose 46-dehydratase (*gmd_1*)	EGZ2997311.1	561	Cytoplasm	Enzymatic action
	Serine recombinase (*PinR*)	WP_000268365.1	549	Cytoplasm	Enzymatic action
	Tyrosine-protein kinase (*wzc*)	WP_000137212.1	2163	Membrane-associated	Enzymatic action
	UTP--glucose-1-phosphate uridylyltransferase (*galF*)	WP_000609087.1	894	Cytoplasm	Enzymatic action
	Chain length determinant protein (*wzzB*)	WP_000027959.1	984	Inner cell membrane	Others (Transport, Metabolism etc.)
	GDP-L-colitose synthase (*colC*)	WP_000866332.1	924	Cytoplasm	Enzymatic action

## Data Availability

The whole genome sequences used in this study were obtained from the National Center for Biotechnology Information (NCBI). Both their accession numbers and the corresponding Assembly accession numbers are listed in the Supplementary Materials.

## Supplementary Materials

The following supporting information can be downloaded at: https://www.mdpi.com/article/10.3390/pathogens14090862/s1, Table S1: Genome assembly details, including GenBank assembly accession numbers, assembly names, strain/isolate designations, virulence genes profile and serotypes; Table S2: serogroup-candidate target markers, including protein name (gene), accession number, amplicon size (bp), number of hits in the target organism, percent coverage, and sequence; Table S3: Further analysis of three discordant isolates.
